# Divergent Mitochondrial Antioxidant Activities and Lung Alveolar Architecture in the Lungs of Rats and Mice at High Altitude

**DOI:** 10.3389/fphys.2018.00311

**Published:** 2018-04-04

**Authors:** Alexandra Jochmans-Lemoine, Susana Revollo, Gabriella Villalpando, Ibana Valverde, Marcelino Gonzales, Sofien Laouafa, Jorge Soliz, Vincent Joseph

**Affiliations:** ^1^Centre de Recherche de l'Institut Universitaire de Cardiologie et de Pneumologie de Québec, Université Laval, Quebec City, QC, Canada; ^2^Instituto Boliviano de Biologia de Altura, Universidad Mayor de San Andrés, La Paz, Bolivia; ^3^Centre National de la Recherche Scientifique, UMR 5023, Université Claude Bernard Lyon 1, Villeurbanne, France

**Keywords:** oxidative stress, mitochondria, high altitude, lung, postnatal hypoxia

## Abstract

Compared with mice, adult rats living at 3,600 m above sea level (SL—La Paz, Bolivia) have high hematocrit, signs of pulmonary hypertension, and low lung volume with reduced alveolar surface area. This phenotype is associated with chronic mountain sickness in humans living at high altitude (HA). We tested the hypothesis that this phenotype is associated with impaired gas exchange and oxidative stress in the lungs. We used rats and mice (3 months old) living at HA (La Paz) and SL (Quebec City, Canada) to measure arterial oxygen saturation under graded levels of hypoxia (by pulse oximetry), the alveolar surface area in lung slices and the activity of pro- (NADPH and xanthine oxidases—NOX and XO) and anti- (superoxide dismutase, and glutathione peroxidase—SOD and GPx) oxidant enzymes in cytosolic and mitochondrial lung protein extracts. HA rats have a lower arterial oxygen saturation and reduced alveolar surface area compared to HA mice and SL rats. Enzymatic activities (NOX, XO, SOD, and GPx) in the cytosol were similar between HA and SL animals, but SOD and GPx activities in the mitochondria were 2–3 times higher in HA vs. SL rats, and only marginally higher in HA mice vs. SL mice. Furthermore, the maximum activity of cytochrome oxidase-c (COX) measured in mitochondrial lung extracts was also 2 times higher in HA rats compared with SL rats, while there was only a small increase in HA mice vs. SL mice. Interestingly, compared with SL controls, alterations in lung morphology are not observed for young rats at HA (15 days after birth), and enzymatic activities are only slightly altered. These results suggest that rats living at HA have a gradual reduction of their alveolar surface area beyond the postnatal period. We can speculate that the elevation of SOD, GPx, and COX activities in the lung mitochondria are not sufficient to compensate for oxidative stress, leading to damage of the lung tissue in rats.

## Introduction

Mammals living at high altitude (HA) can deploy several strategies to counteract ambient hypoxia, and it is noteworthy that different types of genetic adaptations or physiological acclimatization to low ambient O_2_ can be observed. Several studies have focused on rodent (Cheviron et al., 2012; Lau et al., 2017) or lagomorph (Li et al., 2009; Bai et al., 2015) species that are endemic to HA in order to understand the genetic mechanisms and signatures underlying HA adaptation. However, recent migration of lowland native species to HA regions offers unique opportunities to document adaptive strategies that can operate on a much shorter time-frame (Storz et al., 2007; Jochmans-Lemoine et al., 2016). Striking examples of this are found in the HA regions of South-America, where new animal species such as rats or mice have migrated over the past five centuries, following the European conquests (Guenet and Bonhomme, 2003; Storz et al., 2007). We recently described key differences between rats and mice that have been living in La Paz (Bolivia, 3,600 m above sea level—SL) under laboratory conditions for several generations (Jochmans-Lemoine et al., 2015). While mice maintained low hematocrit, high ventilation, and larger lung volumes, rats had high hematocrit, lower ventilation, signs of severe pulmonary hypertension, and lower arterial oxygen saturations when challenged with high or low levels of inspired O_2_. Overall, the phenotype observed in rats is strikingly similar to the pathological features of chronic mountain sickness, a syndrome of de-adaptation to hypoxia with chronic hypoventilation, high hematocrit levels, pulmonary hypertension, and reduced diffusion capacity within the lungs (Julian et al., 2015; Villafuerte and Corante, 2016). We also reported that it was possible to partially revert this phenotype by a simple exposure to an enriched O_2_ environment during the first 2-weeks following birth (Lumbroso et al., 2012). HA rats exposed to inspired SL O_2_ pressure during postnatal development had lower hematocrit, signs of reduced pulmonary pressure, and lungs with reduced airspaces suggesting improved alveolar development.

The large surface area of the lungs and continuous contact of the lung epithelium with environmental O_2_ predispose to oxidative damage (Santus et al., 2014), and oxidative stress in the lung is a potent cause of injury and inflammation (Salama et al., 2014). Furthermore, it is well acknowledged that chronic hypoxia (Turrens, 2003) and HA (Dosek et al., 2007) lead to excessive reactive oxygen species (ROS) production and oxidative stress. A recent study showed that delayed alveolar formation following exposure to postnatal hyperoxia (used as a model of bronchopulmonary dysplasia) is linked to the mitochondrial-dependent generation of ROS (Datta et al., 2015). Accordingly, we performed the present study to assess the hypothesis that the typical low alveolar surface area in the lungs of rats living at HA is associated with impaired gas exchange and oxidative stress in the lungs. We compared arterial oxygen saturation under graded levels of hypoxia, lung histology, and the activity of pro- and anti-oxidant enzymes in homogenized lung tissue from adult rats living at SL or HA. Similar experiments were performed in mice, which represented a control group of animals with successful physiological adaptations to HA. In addition, we tested the hypothesis that altered lung histology and oxidative stress are already present during postnatal development in rats at HA by using newborn rats (2 weeks-old) exposed to HA or SL. Finally, we tested the effects of postnatal hypoxia in rats living at SL, or postnatal O_2_ enrichment in rats living at HA during postnatal days 4–14 (P4–P14—the timeframe corresponding to lung alveolar formation in rats—Burri et al., 1974) on arterial oxygen saturation during graded levels of hypoxia, alveolar morphology and oxidative stress balance.

## Materials and methods

### Animals and experimental groups

#### Animals

This study was carried out in accordance with the recommendations of the Canadian Council of Animal Care. The protocols were approved by the Committee on Animal Care and Use for Laval University in Canada, or by the scientific committee of the Instituto Boliviano de Biologa de Altura (IBBA) in Bolivia. In both countries, animals were housed under standard conditions, had access to food and water ad-libitum, and were exposed to a 12:12 h light/dark cycle.

##### In Canada

Adult Sprague Dawley male rats and FVB mice around 2 months old were ordered from Charles-Rivers (Charles-River—St-Constant, Québec), and left undisturbed at least for 7 days before being used. Newborn rats were obtained from 8 Sprague Dawley female rats (Charles-River—St-Constant, Québec) that were housed with males for mating for at least 7 consecutive days. Once pregnancy was confirmed by weight gain, the females were isolated in a separate cage. At birth, all litters were culled to 12 pups with an equal number of males and females.

##### In Bolivia

All rats were obtained from the Instituto Boliviano de Biologia de Altura (IBBA, La Paz, Bolivia, 3,600 m). These are Sprague-Dawley rats that were originally imported from France (IFFA-CREDO) in 1992, and continuously bred at the IBBA. Males and females were left together until pregnancy was confirmed, and at birth all pups were kept alive (litters generally have less than 12 pups). Adult mice were obtained from the Instituto Nacional de Laboratorios de Salud (INLASA, La Paz, Bolivia). These mice are descended from a lineage of animals that were originally imported from France (IFFA-CREDO) 20–25 years ago and have 73.11% homology with the FVB strain, suggesting that they are either a mix of FVB with 2 other strains or an outbred strain (Jochmans-Lemoine et al., 2015).

#### Exposure to postnatal hypoxia in Canada

Four days after birth, animals were placed under normobaric hypoxia in a 50 L plexiglass chamber, connected to an Oxycycler (A84XOV, BioSpherix, Redfield, NY, USA). On the 1st day of exposure, the O_2_ level in the chamber was progressively decreased to 13.5% (1% O_2_ every 20 min) and kept constant at this level for 10 consecutive days (until postnatal day 14). This corresponds to an inspired PO_2_ around 100 mmHg, which is the inspired PO_2_ found in La Paz. To avoid humidity and CO_2_ accumulation, Drierite (anhydrous calcium sulfate; Hammond Drierite, Xenia, OH, USA) and Amsorb Plus (calcium hydroxide; Armstrong Medical, Coleraine, North Ireland) were placed inside the chamber. Control animals were kept in the same room and left undisturbed (except for normal cage cleaning—once a week).

At postnatal day 14 (P14), the O_2_ level inside the chamber was progressively returned to 21% (at the rate of 1% O_2_ every 15 min), the animals were sacrificed 24 h later for lung tissue sampling.

#### Exposure to postnatal O_2_ enrichment in Bolivia

Four days after birth, animals were placed under hypobaric normoxia in a 50 liter plexiglass chamber or left in ambient room air. The O_2_ level was continuously measured in the chamber, and maintained at ≈ 32% O_2_ (corresponding to an inspired PO_2_ of 160 mmHg, the normal SL inspired PO_2_) by a continuous flow from a calibrated gas tank. The CO_2_ level was controlled twice daily with a dedicated CO_2_ sensor and never exceeded 0.3%. The air inside the chamber was continuously mixed with a small fan, and there was no apparent humidity inside the chamber. At P14, the chamber was opened, and the animals were returned to ambient air. These animals were sacrificed 24 h later for lung tissue sampling.

### Pulse oximetry recordings

Pulse oximetry recordings were performed in sealed chambers (adapted to the size of the animals and constantly flushed with fresh room air). *In Canada*, animals were equipped with a neck collar (Mouse OX® STARR Life Sciences Corp, USA) and *in Bolivia*, animals were equipped with a limb sensor (MouseSTAT—Kent Scientific, Torrington, CT, USA) allowing recordings of pulse oximetry capillary O_2_ saturation (SpO_2_). We have verified that at SL, the two systems gave similar SpO_2_ values under graded hypoxia. Each animal was placed in the chamber for a period of acclimatization (10–15 min), then the inflowing tube was switched to a nitrogen gas line calibrated to obtain the desired O_2_%. *In Canada*, animals were subsequently exposed to 18, 15, 12, and 9% O_2_, each for 10 min and *in Bolivia*, animals were exposed to 32% O_2_–corresponding to the inspired air at SL—followed by 18, 15, 12% O_2_ for 10 min each. In the present work, we report values that have been collected under comparable pressures of inspired O_2_ at 160 mmHg (SL room and 32% O_2_ at HA) and 90 mmHg (12% O_2_ at SL and 18% O_2_ at HA).

### Lung dissection, histology, and morphology

In both countries, we used 2 males from each litter of newborn rats (4 litters per group) and 4 adult males of each species (rats and mice). Animals were deeply anesthetized and perfused through the heart with ice-cold PBS solution. Next, a catheter was fixed in the trachea, the lungs were inflated with 4% PFA for 30 min at a constant pressure of 24 cm H_2_O, then the trachea was ligated and the lungs dissected. The total volume of the inflated lungs was measured by liquid displacement. Dissected lungs were kept in 4% PFA for 24 h at room temperature. The next day, the lungs were separated into left and right lungs (for newborn rats and adult mice) or into 5 lobes (for adult rats), which were automatically embedded in paraffin (*in Canada*, using the Tissue-Tek VIP, Miles scientific) or manually (*in Bolivia* as previously described; Jochmans-Lemoine et al., 2015). Twenty-four hours later, the samples were included in paraffin and stored until they were processed for lung histology as previously described (Jochmans-Lemoine et al., 2015). We have verified the two embedding approaches gave similar morphological results on a sample of SL lung for both species. In another 4 newborn male rats and 6 adult rats and mice, the lungs were immediately removed from the chest after the cardiac PBS perfusion, weighed, frozen on dry ice and stored until they were processed to determine enzymatic activity.

#### Lung morphology

After deparaffinization and coloration (Hematoxylin), images of each lung section were captured (magnification: x100; see Jochmans-Lemoine et al., 2015, for details). We analyzed 3 slides per animal, and randomly selected 3 non-overlapping images from each slide (we used a total of 4 adult male rats and mice in each group, and 8 P15 male rats in each group). The Mean Linear Intercept (Lm) was determined by overlapping a grid of 20 horizontal and vertical lines (189 μm each) on each image and by counting the number of intersections with alveolar walls (Hsia et al., 2010). When a line crossed a vessel wall rather than an alveolar wall it was counted as 0.5 intersections. Lm was calculated by using the following equation: Lm = (N.d)/m, with N being the number of lines (20), d the length of each line (189 μm), and m the number of intersections with alveolar walls. From Lm values, we calculated the relative alveolar surface area as S (m^2^/cm^3^) = 4 V/Lm, with V being the volume of one image (Hsia et al., 2010). An estimation of the total alveolar surface area was calculated as the product of the relative alveolar surface area and lung volume.

To compare lung morphology between adult rats vs. mice, and between P15 rats living at SL vs. HA, we used allometric scaling parameters as previously described (Jochmans-Lemoine et al., 2015), with the corresponding units: lung weight (g/g), lung volume (ml/g), and relative alveolar surface area (m^2^/cm^3^/g^−0.13^).

### Biochemical analysis

#### Protein extraction

Protein was extracted from frozen lungs (50–100 mg) as follows: after homogenization in 1 ml of PBS-EDTA (0.5 mM), the samples were centrifuged for 4 min at 1,500 g and then for 10 min at 12,000 g (all centrifugations at 4°C). Four aliquots (200 μl) of the cytosolic fraction were recovered from the second centrifugation and stored until further analysis. The remaining pellet was homogenized in 1.5 ml of mitochondrial isolation buffer (250 mM sucrose, 1 mM EGTA and 20 mM Tris-base pH 7.3) and centrifuged for 10 min at 1,500 g. We collected the supernatant in a new tube and centrifuged it once more at 9,000 g for 11 min. The supernatant was eliminated and the pellet (containing the mitochondrial protein fraction) homogenized with 300 μl of the mitochondrial isolation buffer and stored at −80°C until further analysis. The concentration of proteins in each fraction was determined using the BCA protein assay kit (ThermoFisher Scientific, catalog #23225) and all subsequent measurements were normalized to protein concentration.

#### Xanthine oxidase (XO) activity

XO activity was assessed in the cytosolic protein fraction using a cocktail containing nitroblue tetrazolium (NTB−2.2 mM in water), Tris-HCl pH 8 (2.8 mM), NaCN 1 mM (to inhibit the degradation of superoxide anions by cytosolic SOD_Cu−Zn_), and diethylene-triamine-penta-acetic acid (DTPA−1.3 mM in Tris-HCl) combined with a fresh solution of hypoxanthine (500 μM per well in Tris-HCl). Fifty microliter of sample, 200 μl of cocktail and 30 μl of hypoxanthine solution was added to each well, and the plate was gently shaken for 40 min at room temperature. The wavelength absorbance of the complex (formazan blue) formed by NTB and the superoxide anion produced by XO in the sample was read at 560 nm every 5 min for 1 h; XO activity corresponded to the slope of the formation of formazan blue by time.

#### NADPH oxidase (NOX) activity

NOX activity was assessed in the cytosolic protein fraction using the same cocktail solution as XO and a fresh solution of NADPH (1 mM). Twenty microliter of sample, 250 μl of cocktail, and 30 μl (100 μM/well) of NADPH were added to each well, the plate was shaken for 2 min at room temperature and absorbance was read at 560 nm every 50 s for 10 min. NOX activity corresponded to the slope of the formation of formazan blue by time.

#### Cytosolic and mitochondrial superoxide dismutase (SOD_Cu−Zn_ and SOD_Mn_)

SOD activity was measured in the cytosolic and mitochondrial protein fractions. SOD_Cu−Zn_ activity was determined in the cytosolic protein fraction by the degree of inhibition of the reaction between the reactive O_2_ anion superoxide (${\text{O}}_{2}\cdot^{-}$: produced by a hypoxanthine-xanthine oxidase system) and NTB. We used the same cocktail described for XO and NOX activities combined with hypoxanthine (0.19 mM) and prepared a fresh solution of xanthine oxidase (1.02 units/ml). We added 20 μl of sample, 250 μl of cocktail and 20 μl of XO to each well, and mixed the plate for 4–5 s at room temperature. The absorbance was quickly read at 450 nm every 50 s for 5 min. SOD_Mn_ activity was similarly determined in the mitochondrial protein fraction. For this assay, 4 wells containing 20 μl of PBS 1X (rather than samples) were used as blanks, and 1 mM of NaCN was added in all wells to inhibit SOD_Cu−Zn_. SOD_Mn_ activity corresponded to the difference between the slopes of the formation of formazan blue by time between the blank and each sample.

#### Glutathione peroxidase (GPx)

GPx activity was determined in the mitochondrial protein fraction as the rate of oxidation of NADPH to NADP+ in a cocktail solution containing glutathione reductase, NADPH (1.7 mM) and reduced glutathione (1.6 mM in water) using H_2_O_2_ (0.036% in water) as a substrate (Paglia and Valentine, 1967). We added 20 μl of sample, 200 μl of PBS 1X, 30 μl of cocktail solution and 30 μl of H_2_O_2_ to each well and mixed the plate for 4–5 s at room temperature. The absorbance was quickly read at 340 nm every 50 s for 5 min. GPx activity was measured as the slope of the NADPH extinction by time.

#### Cytochrome c oxidase assay

Complex IV is the last enzyme of the mitochondrial electron transport chain and catalyzes oxidation of the reduced cytochrome c by O_2_. We measured the maximum activity of cytochrome c oxidase (COX—complex IV of the mitochondrial respiratory chain) by measuring O_2_ consumption in the mitochondrial protein fraction using a high-resolution respirometer system (Oroboros oxygraph-2k). We used 50–150 μl of sample, with 1 μl of Antimycin A, 5 μl of ascorbate and 5 μl of Tétraméthyl-paraphénylènediamine (TMPD) in 2 ml of mitochondrial respiratory buffer (0.5 mM EGTA, 3 mM MgCl_2_, 60 mM potassium lactobionate, 10 mM KH_2_PO_4_, 20 mM Hepes, 110 mM sucrose, 1 g/l BSA). The maximal activity of COX was read when O_2_ consumption rate was stable (typically a few minutes after starting the recording).

### Statistical analysis

All values are reported as mean ± s.e.m., and the significant *P*-value was set at 0.05. We used GraphPad Prism 6.0c for all analysis. We analyzed arterial oxygen saturation at 160 mmHg and 90 mmHg, lung morphology and the protein assays using a two-way ANOVA with species and altitude as grouping variables. When significant effects or a significant interaction between species and PiO_2_ appeared, a *post-hoc* analysis was performed (Fisher's LSD).

*P*-values are reported in the figures with the following general pattern: ^*^, ^**^, ^***^, and ^****^ are used to report *P* < 0.05, 0.01, 0.001, and 0.0001, respectively. Main ANOVA results are reported in the text whereas Ficher's LSD results are found in the figures.

## Results

### Lung architecture is preserved at HA in adult mice but impaired in rats

**Figure 1:**
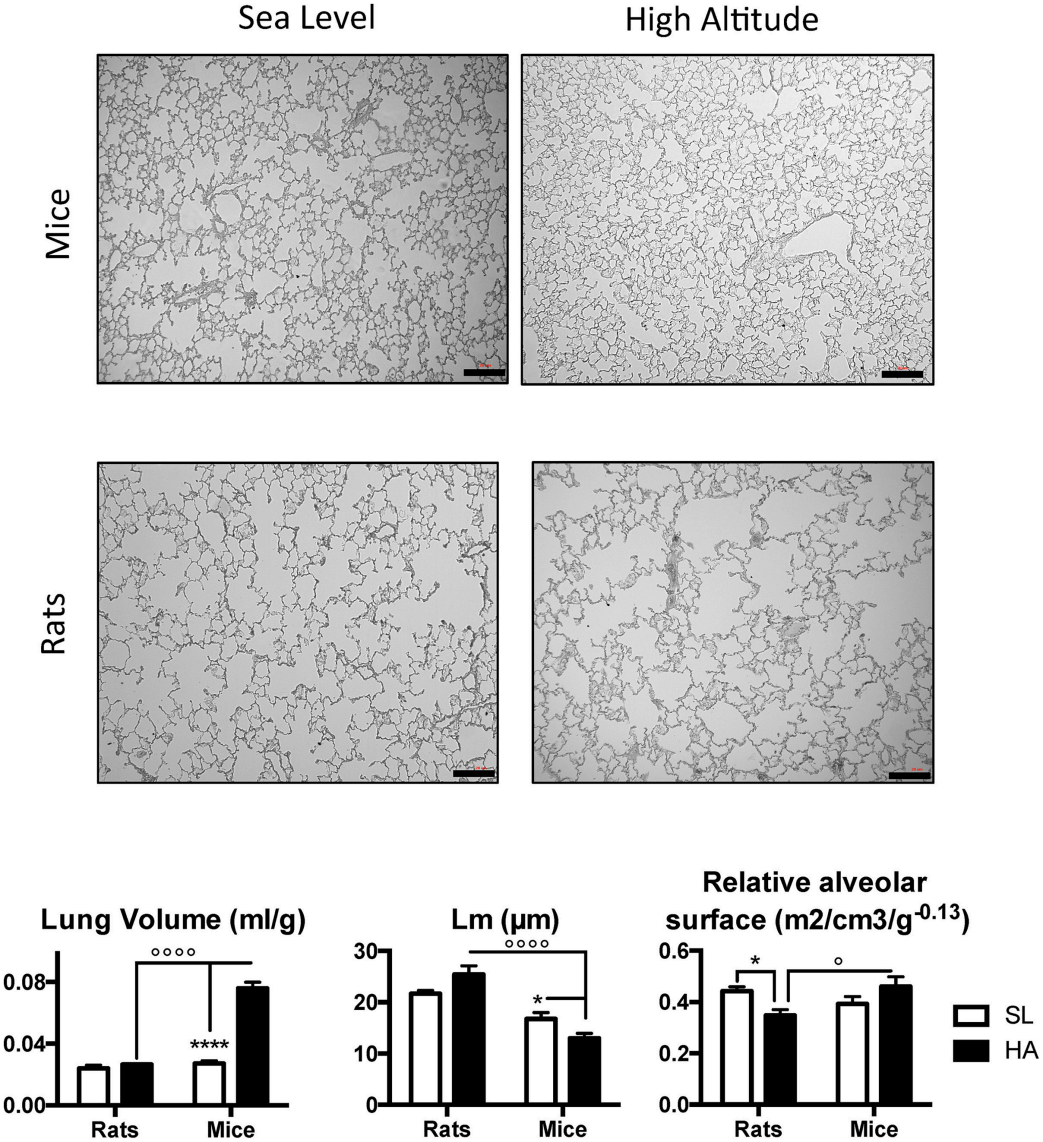
Representative microphotographs showing lung and alveolar structures in adult rats and mice living at sea level (SL—Quebec-city, Canada), and at high altitude (HA—La Paz, Bolivia, 3,600 m). Lower panels show lung volume, mean linear intercept (Lm), and relative alveolar surface in rats and mice at SL and HA. All values are mean + s.e.m. Number of animals in each group: *n* = 4. Scale bars: 50 μm. ^*^P < 0.05, and ^****^P < 0.0001 HA vs. SL. ^◦^*P* < 0.05, and ^◦◦◦◦^*P* < 0.0001 mice vs. rats.

Representative images of lung slices in adult rats and mice living at SL and HA are presented in Figure 1, along with the results of the morphometric analysis. Adult mice living at HA have significantly increased (over 3-fold) lung volume compared with their SL counterparts, and this is not observed in rats (*P*-value for altitude <0.0001, *P*-value for altitude x species <0.0001). The mean linear intercept (Lm) was significantly lower in mice living at HA compared with SL and tended to be higher in rats living at HA compared with SL (*P*-value for species x altitude = 0.01—*post-hoc P*-value for altitude in rats = 0.067). Comparisons between HA and SL revealed that rats had a reduced mass-corrected relative alveolar surface area (in m^2^/cm^3^/g^−0.13^) at HA; but when compared with mice at HA, the mass-corrected relative alveolar surface area was lower in rats (*P*-value for species x altitude = 0.02). These results suggest that the lung morphology of HA rats does not facilitate improved gas exchange.

### HA mice maintain a higher arterial oxygen saturation in response to graded hypoxia

**Figure 2:**
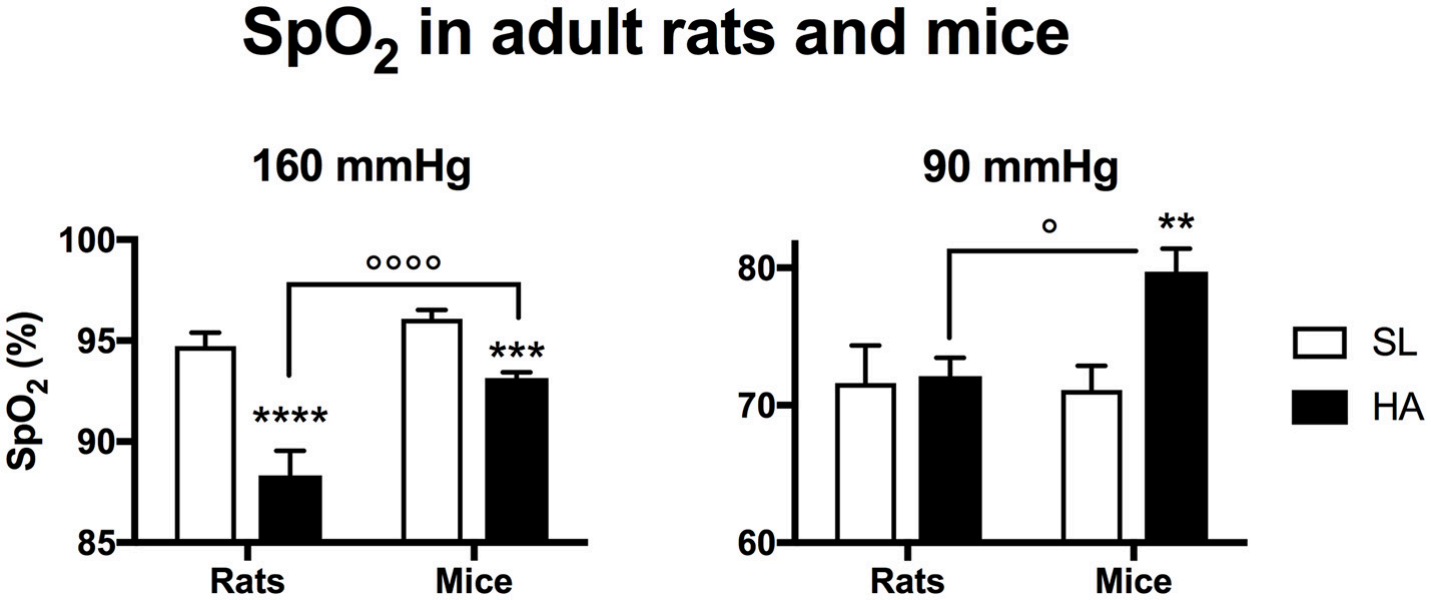
Arterial O_2_ saturation recorded in adult rats and mice living at sea level (SL—Quebec-city, Canada), and at high altitude (HA—La Paz, Bolivia, 3,600 m) during exposure to 160 or 90 mmHg. All values are mean + s.e.m. Number of animals in each group at SL: *n* = 10 mice, 6 rats. Number of animals in each group at HA: *n* = 9 mice, 6 rats. ^**^P < 0.01, ^***^P < 0.001, and ^****^ *P* < 0.0001 HA vs. SL. ^◦^*P* < 0.05, and ^◦◦◦◦^*P* < 0.0001 mice vs. rats.

In rats, SpO_2_ was lower at HA than at SL for normoxic O_2_ levels (160 mmHg), but was similar between the two altitudes for a PiO_2_ of 90 mmHg (Figure 2). In mice at HA, SpO_2_ was higher than HA rats under PiO_2_'s of 160 and 90 mmHg. At 90 mmHg, HA mice had a significantly higher SpO_2_ than SL mice.

### Mitochondrial antioxidant activities are increased in the lungs of adult rats at HA

**Figure 3:**
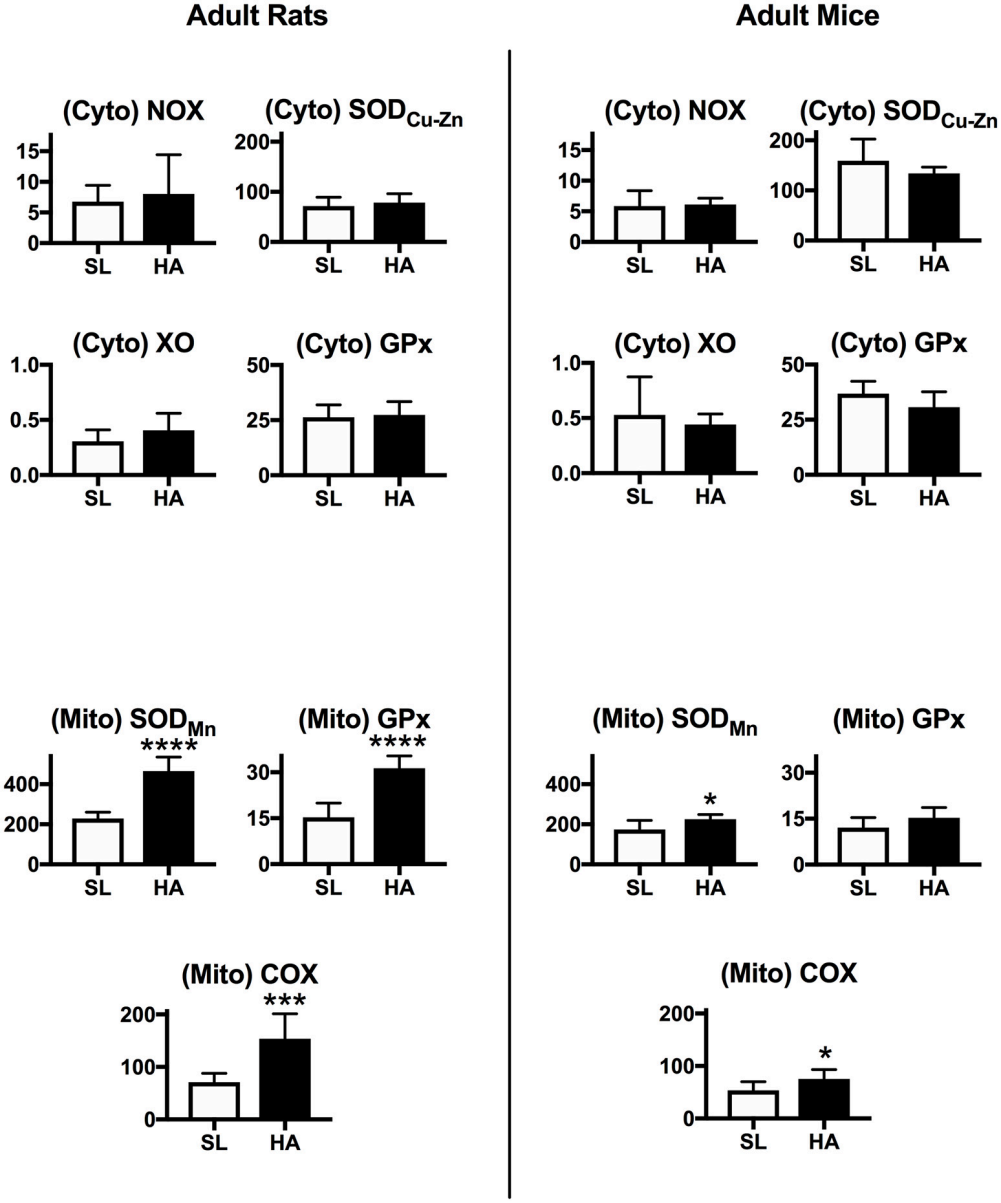
**Upper panels:** Activity of NADPH oxidase (NOX), xanthine oxidase (XO), copper-zinc superoxide dismutase (SOD_Cu−Zn_), and glutathione peroxidase (GPx) measured in cytosolic (Cyto) lung extracts. **Lower panels**: Activity of manganese superoxide dismutase (SOD_Mn_), glutathione peroxidase (GPx), and cytochrome-c oxidase (COX) measured in mitochondrial (Mito) lung extracts in rats and mice living at sea level (SL), or high altitude (HA). All values are mean + s.e.m. Values for NOX, XO, GPx, and SOD are in nmol/min/μg protein, and for COX in pmol O_2_/sec/mg protein (see text for further details). Number of animals in each group: SL rats and mice *n* = 9 and 10, HA rats and mice *n* = 6 and 6. ^*^P < 0.05, ^***^P < 0.001, and ^****^P < 0.0001. HA vs. SL.

The activities of NOX, XO, GPx, and SOD_Cu−Zn_ in the cytosolic protein fraction from the lungs of adult rats and mice are similar between SL and HA (Figure 3). In the mitochondrial protein fraction, there was increased SOD_Mn_ and GPx activity (around 2-fold) in HA adult rats compared with SL. The activity of Complex IV of the mitochondrial respiratory chain (or cytochrome c oxidase—COX) was also increased in rats living at HA compared with SL. In mice, the activity of mitochondrial SOD_Mn_ and COX were slightly increased at HA compared with SL. Overall this suggests that important changes in mitochondrial oxidative reactions occur in the lungs of rats living at HA compared with SL control animals, while this does not occur in mice.

### The alveolar surface area in young rats is impaired by postnatal hypoxia at SL, but preserved at HA

**Figure 4:**
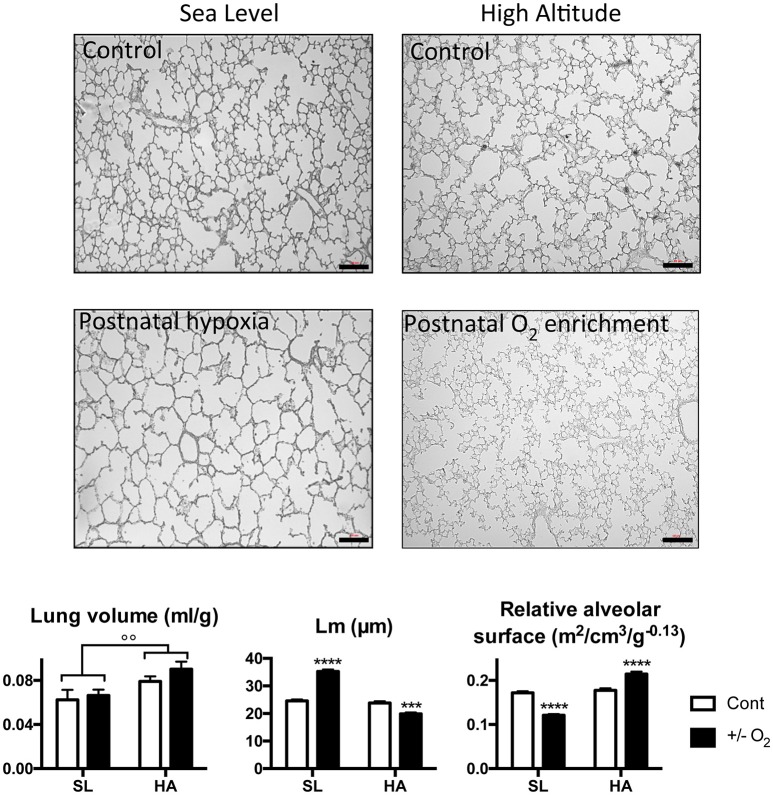
Representative microphotographs showing lung and alveolar structures in young (postnatal day 15) rats living at sea level (SL—Quebec-city, Canada), and at high altitude (HA—La Paz, Bolivia, 3,600 m), following exposure to ambient O_2_ level (control), hypoxia (at SL−13.5% O_2_), or O_2_ enrichment (at HA−32% O_2_) between postnatal days 4–14. Lower panels show lung volume, mean linear intercept (Lm), and relative alveolar surface in control (Cont) rats and in rats exposed to hypoxia (at SL), or normoxia (at HA). All values are mean + s.e.m. Number of animals in each group: SL control and hypoxic rats *n* = 9 and 12, HA control and O_2_ enriched rats *n* = 8 and 8. Scale bars: 50 μm. ^***^P < 0.001, and ^****^P < 0.0001 ± O_2_ vs. Cont. ^◦◦^*P* < 0.01 HA vs. SL.

Representative images of lung slices along with the results of the morphometric analysis in SL and HA P15 rats exposed to room air, postnatal hypoxia (at SL), or postnatal O_2_ enrichment (at HA) are presented in Figure 4. At P15, SL rats exposed to postnatal hypoxia have a similar lung volume to control rats, but a higher value of Lm and reduced mass-corrected relative alveolar surface area. At HA, P15 rats have higher lung volumes compared with SL P15 rats, and a similar Lm and alveolar surface area. At HA, postnatal O_2_ enrichment reduced Lm and increased the mass-corrected relative alveolar surface area.

### Arterial oxygen saturation in young rats is impaired by postnatal exposure to hypoxia at SL

**Figure 5:**
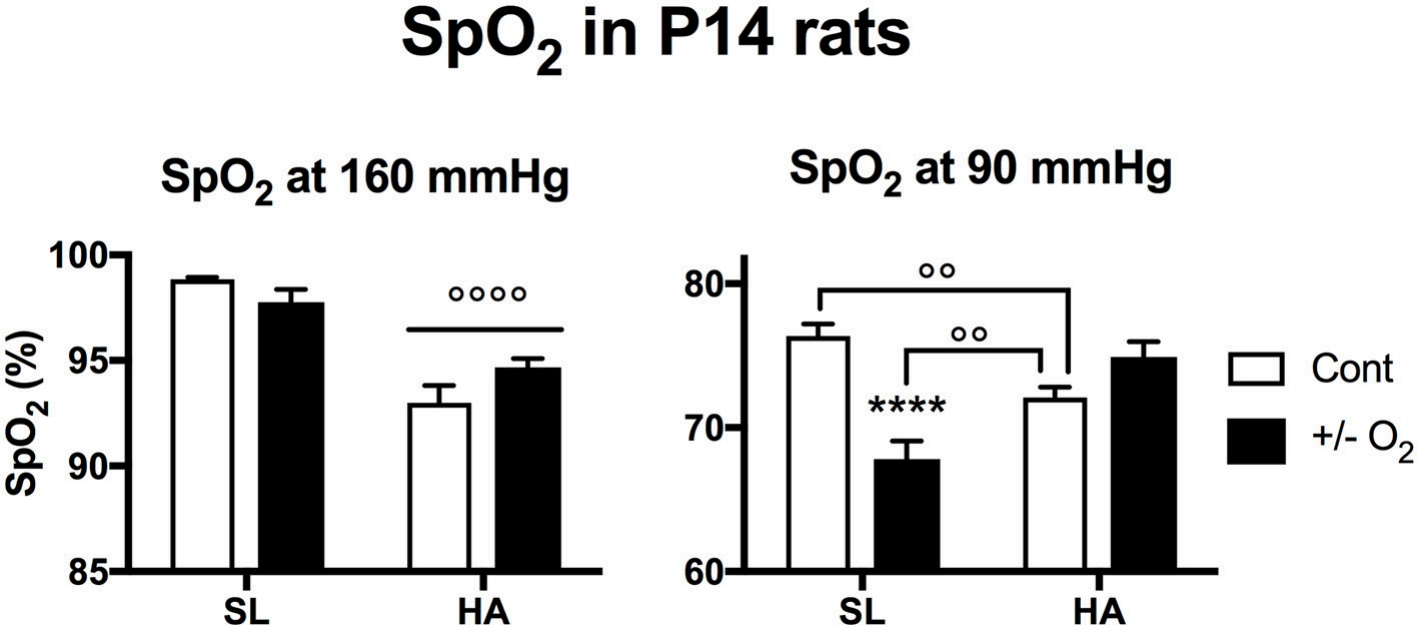
Arterial O_2_ saturation recorded in young (postnatal day 14) rats living at sea level (SL—Quebec-city, Canada), and at high altitude (HA—La Paz, Bolivia, 3,600 m), during exposure to ambient O_2_ levels (control), hypoxia (at SL−13.5% O_2_), or O_2_ enrichment (at HA−32% O_2_) between postnatal days 4–14. All values are mean + s.e.m. Number of animals in each group at SL: control and hypoxic rats *n* = 15 and 17, and at HA: control and O_2_ enriched rats *n* = 10 and 12. ^****^P < 0.0001 ± O_2_ vs. Cont. ^◦◦^*P* < 0.01, and ^◦◦◦◦^*P* < 0.0001 HA vs. SL.

At SL, postnatal exposure to hypoxia significantly reduced SpO_2_ in young rats during a PiO_2_ of 90 mmHg (Figure 5). Interestingly, when young control rats maintained at HA were exposed to 90 mmHg, the observed SpO_2_ was lower than for control SL rats, but was higher than SL rats exposed to postnatal hypoxia.

### Cytosolic SOD activity in the lungs is reduced by postnatal hypoxia at SL, but increased by postnatal O_2_ enrichment at HA

**Figure 6:**
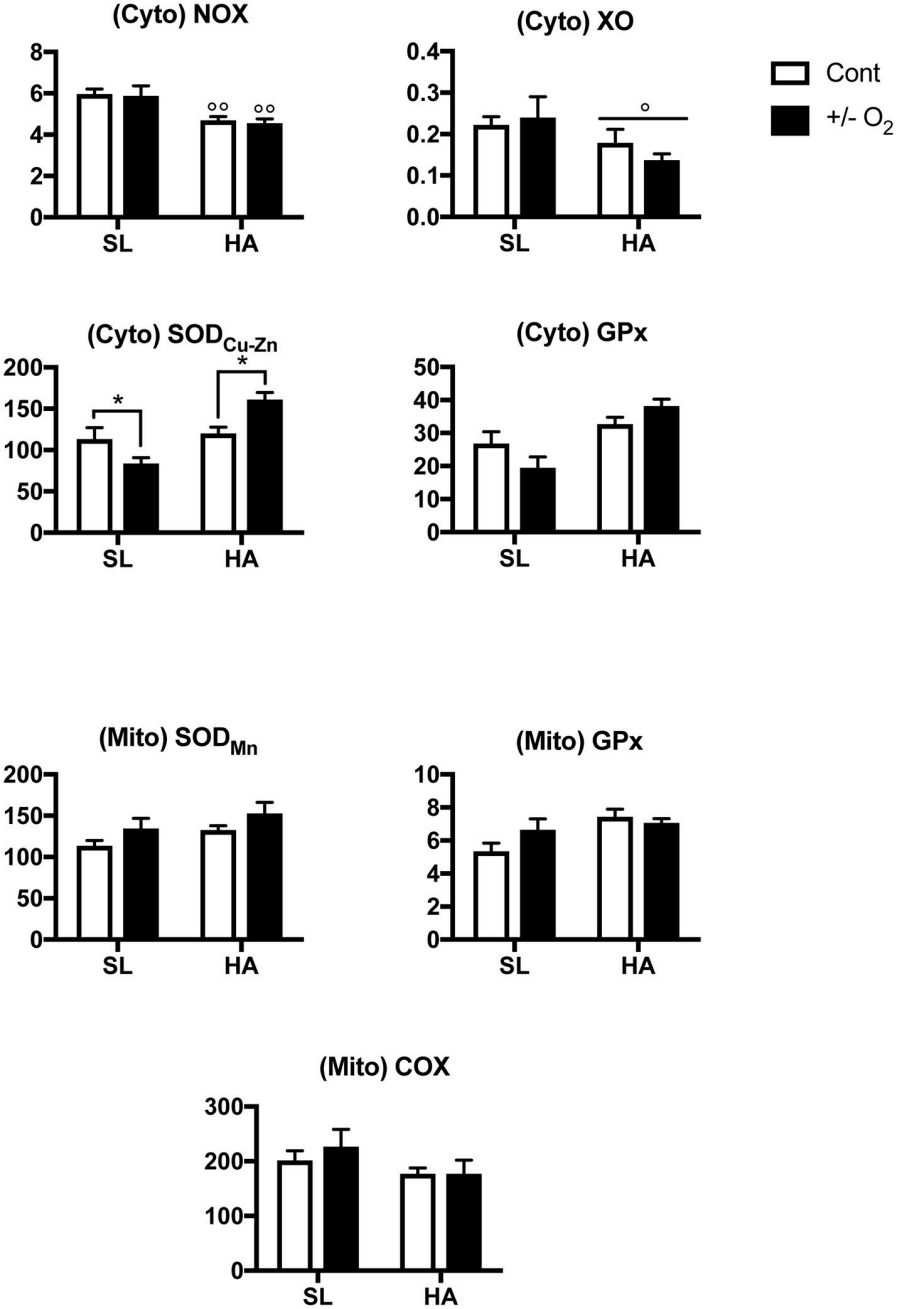
**Upper panels:** Activity of NADPH oxidase (NOX), xanthine oxidase (XO), copper-zinc superoxide dismutase (SOD_Cu−Zn_), and glutathione peroxidase (GPx) measured in cytosolic (Cyto) lung extracts. **Lower panels**: Activity of manganese superoxide dismutase (SOD_Mn_), glutathione peroxidase (GPx), and cytochrome-c oxidase (COX) measured in mitochondrial (Mito) lung extracts in young (postnatal day 15) rats living at sea level (SL—Quebec-city, Canada), and at high altitude (HA—La Paz, Bolivia, 3,600 m), following exposure to ambient O_2_ level (control), hypoxia (at SL−13.5% O_2_), or O_2_ enrichment (at HA−32% O_2_) between postnatal days 4–14. All values are mean + s.e.m. Values for NOX, XO, GPx, and SOD are in nmol/min/μg protein, and for COX in pmol O_2_/sec/mg protein (see text for further details). Number of animals in each group at SL: control and hypoxic rats *n* = 9 and 8, and at HA: control and O_2_ enrichment rats *n* = 8 and 6. ^*^P < 0.05 ± O_2_ vs. Cont. ^◦^*P* < 0.05, and ^◦◦^*P* < 0.01 HA vs. SL.

NOX and XO enzymatic activities were lower at HA than at SL (*P*-value for altitude = 0.0003 and 0.038 respectively—Figure 6), but there was no effect resulting from exposure to postnatal hypoxia (at SL), or O_2_ enrichment (at HA). At SL, exposure to postnatal hypoxia significantly reduced SOD activity, while at HA postnatal O_2_ enrichment had the opposite effect (*P*-value for altitude × O_2_ level = 0.002). These results suggest reduced production of ROS by NADPH, and greater anti-oxidant capacity by GPx in P15 control HA rats compared with SL controls. In the mitochondrial protein fraction, there was no change in enzyme activities (Figure 6) due to postnatal O_2_ level or altitude.

## Discussion

In the present study, we reported that compared to SL controls, in mice, exposure to HA for several generations led to larger lungs with a higher relative alveolar surface area. The opposite response was observed in rats: at HA, lung volume was not increased compared with SL, and the relative alveolar surface area was reduced. In line with this, mice exposed to low O_2_ levels at HA had higher SpO_2_ values than rats, but remarkably HA mice also had higher SpO_2_ values than SL mice, indicating highly efficient gas exchange functions. Because previous studies demonstrated that chronic hypoxia (Turrens, 2003) and HA (Dosek et al., 2007) lead to excessive ROS production and oxidative stress, a well-known cause of lung injury and inflammation (Salama et al., 2014), we hypothesized that such damage could occur in rats. We measured the activities of pro- and anti-oxidant enzymes in cytosolic and mitochondrial protein extracts in the lungs. Our data showed that HA had no effect on the activity of pro- or anti-oxidant enzymes in the cytosol. However, antioxidant enzyme activities in the mitochondrial protein fraction were about 2 times higher in HA rats compared with SL rats, whereas in mice there was a smaller increase only for the SOD_Mn_ and COX activities (1.25 times higher).

Several studies have shown that postnatal hypoxia reduced lung alveolarization in rats (Blanco et al., 1991; Truog et al., 2008); we thus tested the hypothesis that at HA, young rats (P15) would present early signs of altered lung alveolarization. Surprisingly, this was not the case as our data showed that at HA, young rats had lung volumes, Lm, and relative alveolar surface areas similar to SL young rats. Interestingly, SpO_2_ values during a PiO_2_ of 90 mmHg were higher in control rats at HA than in SL rats exposed to postnatal hypoxia, therefore indicating more efficient gas exchange function at HA in the former group. Furthermore, our data indicated that O_2_ enrichment between postnatal days 4–14 at HA can further increase the alveolar surface area at P15, but this was not associated with improved SpO_2_ values. In previous studies, however, we showed that at HA, O_2_ enrichment during postnatal development has long-term effects including improved lung alveolar architecture, reduced pulmonary hypertension and lower hematocrit in adult rats (Lumbroso et al., 2012), likely indicating long-term improvement in arterial O_2_ saturation. Young rats at HA had lower activity of NADPH and XO (two major sources of cytosolic ROS production) compared with SL rats, while O_2_ enrichment during postnatal development increased SOD activity in the cytosol, and hypoxic exposure during the same period at SL had the opposite effect.

Overall, these findings suggest that in adult HA rats, the high activities of SOD, GPx, and COX could be a strategy to buffer excessive mitochondrial ROS production. In contrast, antioxidant enzyme activities are increased in young HA rats, and pro-oxidant enzymes are concurrently decreased as a strategy to maintain ROS equilibrium.

### Alterations of lung morphology are present in adult rats at HA despite high mitochondrial anti-oxidant activity

Hypoxic exposure induces ROS production in the mitochondria (Jezek and Hlavata, 2005), and excessive ROS production induces pro-inflammatory responses and has cytotoxic effects including lipid membrane peroxidation, DNA damage, oxidation of amino acids and oxidative cleavage of peptide bonds in proteins. A previous study showed that iron supplementation induced lung injuries in rats at HA through excessive production of ROS and induction of inflammatory responses (Salama et al., 2014). Moreover, a role for oxidative stress has been proposed in the development of chronic obstructive lung diseases (van Eeden and Sin, 2013). Since adult rats at HA had elevated activity of mitochondrial antioxidant enzymes, we can hypothesize that there was elevated ROS production in the mitochondria of lung cells in rats, but not mice. Indeed, even if ROS can be produced in cells by diverse mechanisms and in several compartments, up to 90% are produced at complexes I and III of the electron transfer chain in mitochondria (Adam-Vizi, 2005). Due to the fact that ROS play a key role in lung diseases, it is possible that the elevated anti-oxidant activity we observed in the lungs of rats living at HA is not sufficient to completely protect against oxidative stress, and this could in part explain why HA rats had low alveolar surface areas. Using the same line of evidence, this implies that mice at HA were not subjected to excessive mitochondrial oxidative stress in the lung, and while we cannot infer a direct causal link, it is striking to observe that in parallel mice had increased relative alveolar surface area.

Cytochrome-c oxidase (COX—complex IV of the electron transfer chain) catalyzes the final step of the electron transport chain and the production of H_2_O from O_2_, thus accounting for mitochondrial O_2_ consumption. COX activity is also important for mitochondrial ROS production, and it is well established that low COX activity, which increases mitochondrial ROS production by complexes I and III of the electron transfer chain, is involved in several pathological conditions (Srinivasan and Avadhani, 2012). Our results showed higher maximum COX activity in rats living at HA than at SL, while this effect of altitude residence was minimal in mice. This is surprising because acute hypoxic exposure reduces COX activity, directly contributing to increasing ROS production in hypoxia (Prabu et al., 2006; Fukuda et al., 2007; Srinivasan and Avadhani, 2012). In the cardiac myocytes of bar-headed geese (a bird species adapted to flight at extreme altitude), maximum COX activity is reduced, but the affinity of COX for reduced cytochrome-c is higher (Scott et al., 2011). A higher affinity for substrates could ensure higher COX activity at a low or normal concentration of reduced cytochrome-c, thus allowing lower ROS production in the electron transport chain (Scott et al., 2011; Cheviron and Brumfield, 2012). Similarly, increased activity of COX was associated with hypoxia tolerance in Tibetan locusts (Zhang et al., 2013). We can, therefore, hypothesize that in HA rats that have been bred at 3,600 m above SL for 25 years, the increased COX activity could contribute to reduced mitochondrial ROS production, which would be in line with the increased SOD_Mn_ and mitochondrial GPx activities. However, these increased enzymatic activities are apparently not sufficient to buffer the deleterious consequences of oxidative stress, leading to pathological responses such as the altered architecture observed in the lungs of HA rats.

**Figure 7:**
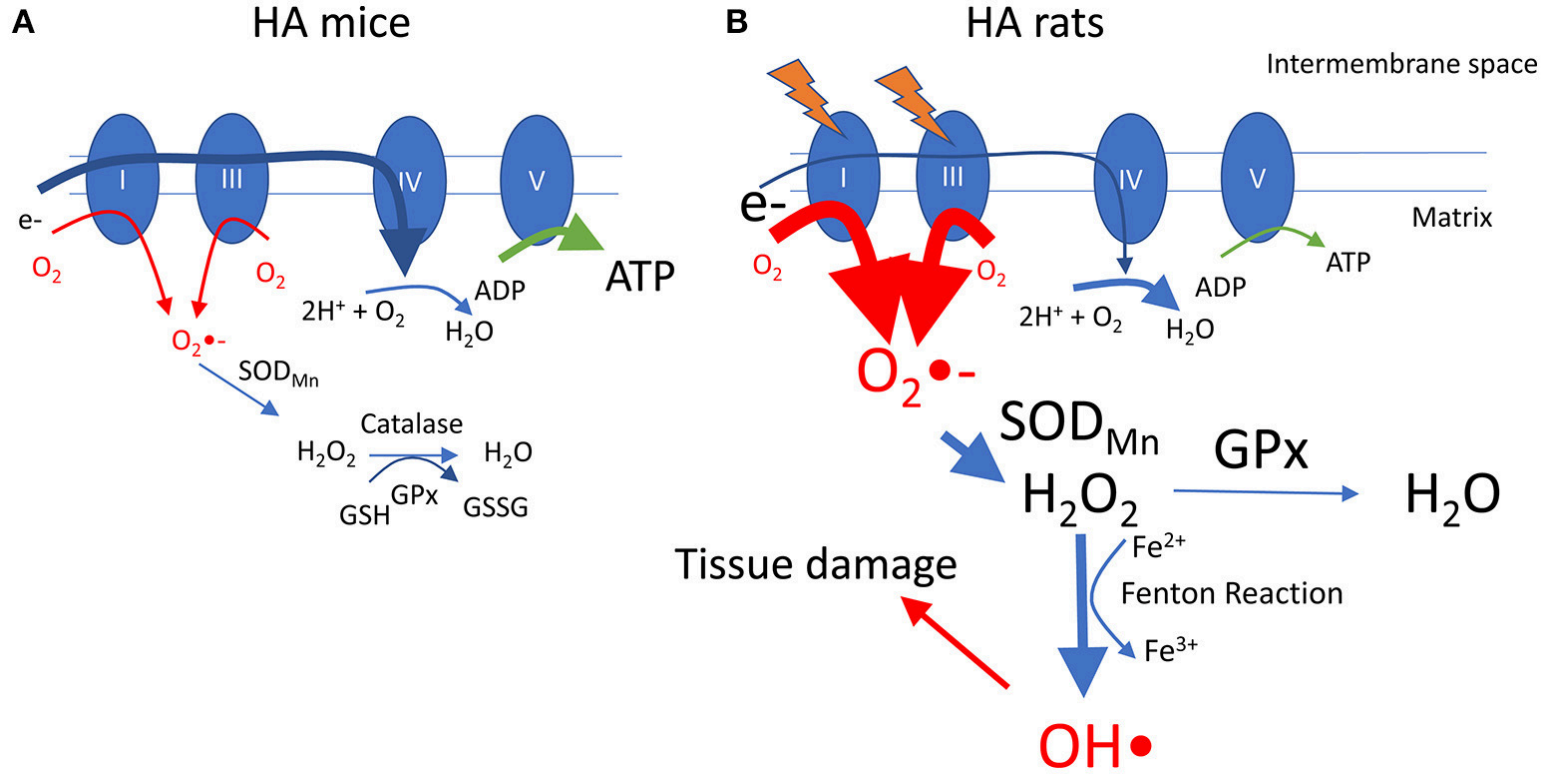
Hypothetical model showing differences between adult rats and mice at HA. In mice **(A)**, the transfer of electrons in mitochondrial complexes is normal (for clarity electron supply is shown only at complex I through oxidation of NADH), resulting in normal production of superoxide (O_2_•-), that can be reduced first to hydrogen peroxide (H_2_O_2_) and then to water by SOD_Mn_ and GPx. In rats **(B)**, exposure to HA alters mitochondrial functions and reduces the activity of complexes I and/or III, leading to a reduced flow of electrons that then become available to generate O_2_•-. High activity of mitochondrial SOD and GPx helps to reduce O_2_•- to H_2_O_2_ and water, but high levels of H_2_O_2_ leads to accumulation of hydroxyl radicals (OH•) through the Fenton reaction. OH•, being a highly reactive ROS, causes accumulation of oxidative damage in the lung tissue, altering the alveolar architecture. High activity of complex IV might contribute to accelerate electron transfer and avoid superoxide accumulation. See text for references.

A summary of these findings is presented in Figure 7, incorporating a hypothetical mechanism underlying our results. Interestingly, we previously reported that, compared with HA mice, HA rats have lower whole body O_2_ consumption and higher glycolytic metabolism as reported by an elevated respiratory exchange ratio (Jochmans-Lemoine et al., 2015). Translated at the cellular level, it is possible to speculate that mitochondrial O_2_ consumption and ATP production are lower in HA rats compared with mice. A state of low mitochondrial respiration increases the production of superoxide anion (O_2_•-) by complexes I and III of the mitochondrial electron transport chain (Murphy, 2009), and in HA rats this can be partially overcome by the high activities of mitochondrial SOD_Mn_ and GPx that reduce O_2_•- to H_2_O_2_ and water. High levels of H_2_O_2_, however, would lead to the production of hydroxyl radicals (HO•) through the Fenton reaction (Thomas et al., 2009), and this highly reactive ROS could interact with biomolecules to cause oxidative damage (Halliwell, 2001). As alveolar lung morphology is altered in rats at HA, we hypothesize that the elevated antioxidant activity is not sufficient to abolish oxidative stress.

### Alteration of lung morphology and elevated mitochondrial anti-oxidant activity are not present in young rats at HA

The fact that HA rats at P15 had a similar Lm and relative alveolar surface area as SL rats was a surprising finding, and was in contrast with the effect of postnatal hypoxia in rats at SL that induced a delay in alveolar formation (the latter results being in line with previous data; Blanco et al., 1991; Truog et al., 2008). In addition, when young rats were exposed to low O_2_, SpO_2_ was higher in HA rats than in SL rats that had been exposed to postnatal hypoxia. Therefore, our results suggest that young HA rats were able to acquire mechanisms to maintain alveolar development in the lungs similar to that of SL rats, and this might have beneficial long-term effects. Indeed, we reported that exposure of HA rats to SL O_2_ levels between postnatal days 4–14 induces a persistent increase in alveolar surface area for adults (Lumbroso et al., 2012), and we have unpublished observations indicating that the alterations induced by postnatal hypoxia at SL persist in adult rats.

We previously reported that the physiological responses observed in rats at HA could be correlated with symptoms reported in chronic mountain sickness (Lumbroso et al., 2012; Jochmans-Lemoine et al., 2015). Interestingly, it has been suggested that chronic mountain sickness is linked to altered perinatal oxygenation (Moore et al., 2007; Julian et al., 2015), and that excessive erythrocytosis (a preclinical state of chronic mountain sickness) at HA is associated with elevated oxidative stress (Julian et al., 2013). Contrasting with the results for adult rats, in young HA rats the cytosolic activity of the pro-oxidant enzymes (NOX and XO) is decreased compared with SL rats. These findings are interesting, because chronic pulmonary hypertension in newborn rats exposed to hypoxia has been associated with ROS generated by xanthine oxidase in the lungs (Jankov et al., 2008). Furthermore, in a study where young mice were exposed to hyperoxia for 72 h between postnatal days 0–3, developmental defects in the lungs were observed at P15 and linked to an exaggerated mitochondrial oxidative stress response and amplification of ROS signaling via NOX-1 (a member of the NOX family of NADPH oxidases; Bedard and Krause, 2007) activity (Datta et al., 2015). Thus, it might be possible that the reduced activity of NOX is in part responsible for the maintenance of lung architecture observed in HA young rats compared with those at SL.

### Study limitations

One of the potential limitations of these studies is the particular nature of the HA species and the fact that they have been genetically isolated for several generations. Aside from the overwhelming influence of constant hypoxic exposure, we cannot completely rule out that these animals have been subjected to genetic drift resulting in a specific phenotype. However, because Charles-River maintains a program to avoid genetic drift between their different stocks of SD rats, we can assume that the original breeders of the HA population and the SD rats used in Quebec are similar. A second limitation is that newborn rats at SL or HA were exposed to room air for 24 h before sacrifice and tissue harvesting. While it is highly unlikely that this short time frame alters lung morphology, it is still possible that it might affect the biochemical analysis, and could contribute to discrepancies in some of our results. As such, the results of the biochemical analysis obtained when animals were exposed to hypoxia at SL or enriched oxygen at HA should be considered cautiously. However, it is striking that there are only limited differences for NOX and XO between newborn rats at SL and HA, while differences are much more substantial in adults. Finally, one should keep in mind that, unfortunately, samples from female rats were not included in these studies. However, despite the fact that ovarian steroids could have potent anti-oxidant effects in mitochondria (Laouafa et al., 2017), we have not detected sex-specific alterations in lung morphology in HA rats (Jochmans-Lemoine et al., 2015).

## Conclusion

We conclude from this study that rats living at HA present an unusually elevated mitochondrial anti-oxidant capacity in the lungs, possibly as a compensatory response to high mitochondrial ROS production. Rats also presented morphological alterations in the lung tissue and a low alveolar surface area—a phenotype known to be induced by high oxidative stress in the lungs. Therefore, our data suggest that the high activities of antioxidant enzymes might not be efficient to adequately protect the lungs. Mice at HA had normal anti-oxidant enzymatic activities in the lungs and were able to develop a higher lung volume, alveolar surface area, and high arterial O_2_ saturation at HA.

Since mitochondrial anti-oxidant enzyme activities and alveolar surface area were normal in young rats at HA, alteration of lung alveolar morphology and oxidative stress balance occur beyond the second postnatal week in rats living at HA. Finally, the phenotype reported in HA rats is strikingly similar to what is observed in patients affected by chronic mountain sickness (Julian et al., 2015; Villafuerte and Corante, 2016). The present findings might thus have clinical relevance for HA residents and provide evidence that targeting mitochondrial ROS production with selective mitochondrial anti-oxidant agents could be an interesting therapeutic opportunity.

## Author contributions

AJ-L, GV, IV, MG: Contributed to sampling and processing tissue samples in Bolivia. AJ-L, SR, SL: Contributed to sampling and processing tissue samples in Canada. AJ-L, JS, VJ: Contributed to initial study design. AJ-L, VJ: Analyzed data, drafted the figures and manuscript, wrote and edited the manuscript. All authors: Commented early drafts of the paper and data presentation, read and approved the final version.

### Conflict of interest statement

The authors declare that the research was conducted in the absence of any commercial or financial relationships that could be construed as a potential conflict of interest.
